# Egg Retrieval as a Cognitive Indicator in Cuckoo Hosts

**DOI:** 10.1002/ece3.70904

**Published:** 2025-01-31

**Authors:** Guo Zhong, Guixia Wan, Longwu Wang, Wei Liang

**Affiliations:** ^1^ Ministry of Education Key Laboratory for Ecology of Tropical Islands, College of Life Sciences Hainan Normal University Haikou China; ^2^ School of Life Sciences Guizhou Normal University Guiyang China; ^3^ Jilin Engineering Laboratory for Avian Ecology and Conservation Genetics, School of Life Sciences Northeast Normal University Changchun China

**Keywords:** avian brood parasitism, egg rejection, egg retrieval, motivation conflict, trade‐off

## Abstract

Egg retrieval behavior in hosts within avian brood parasitism systems was found to be regulated by the motivation to reject parasitic eggs. However, due to the limitations in the research systems, there is a lack of effective validation regarding the adaptation mechanisms of cuckoo hosts to the conflict between retrieving their own eggs outside the nest and rejecting parasitic eggs. This study uses Daurian redstarts ( *Phoenicurus auroreus* ), a secondary cavity‐nesting host parasitized by common cuckoos ( *Cuculus canorus* ), to verify the adaptive decision‐making of the host between egg retrieval and egg rejection by simulating the occurrence of eggs outside the nest. The results showed that Daurian redstarts ignored 60.6% of highly mimetic conspecific eggs, with a retrieval rate of only 18.2%. Additionally, Daurian redstarts rejected 21.2% of conspecific eggs. However, non‐mimetic budgerigar ( *Melopsittacus undulatus* ) and white model eggs were more likely to be directly rejected (75% and 86.4%, respectively) with no retrieval events. Our findings suggest that egg retrieval behavior in Daurian redstarts is likely influenced by the cognitive process of rejecting parasitic eggs, leading to occasional over‐identification and difficulty in decision‐making between egg retrieval and egg rejection, especially in the context of conflicting motivations.

## Graphical abstract

Little work on the cuckoo hosts adapting mechanism to the conflict between ignoring own eggs and rejecting parasitic eggs. The Daurian redstart ( *Phoenicurus auroreus* ), a cuckoo host, ignored most conspecific eggs with only a retrieval rate of 18.2%, while it rejected most non‐mimetic eggs with no egg retrieval. This is the first study showing that the behavior of ignoring eggs outside the nest in cuckoo hosts is related to egg recognition abilities and egg retrieval could be a cognitive indicator.

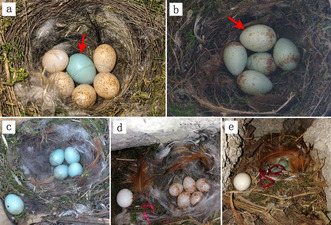

## 1 Introduction

The correct retrieval of their own eggs from the vicinity of the nest back into the nest is considered a reproductive strategy for most ground‐nesting birds to reduce offspring loss (Poulsen 1953; Petit, Petit, and Petit 1989; Lank et al. 1991). The studies suggest that this behavior may also be common among secondary cavity‐nests passerines, particularly among hosts parasitized by obligate brood parasites such as the common cuckoo ( *Cuculus canorus* ) and brown‐headed cowbird ( *Molothrus ater* ) (Yang, Liang, and Møller 2019; Yang et al. 2024; Yang and Zhang 2024; Peer and Vozza 2024). For instance, in the ongoing host‐cuckoo arms race, mislaid parasitic egg laying outside the nest cup, as well as the egg‐evicting behavior of hatchling cuckoos, may be the main drivers for the evolution of egg recognition and retrieval behaviors in hosts. In fact, the retrieval of their own eggs versus the rejection of parasitic eggs likely represents two conflicting motivations (Yang, Liang, and Møller 2019; Yang and Zhang 2024; Peer and Vozza 2024). However, due to the limitations of the research system (Lyon and Shizuka 2017; Liu et al. 2023; Peer and Vozza 2024; Yang et al. 2024; Zhang et al. 2024), the decision‐making mechanisms of hosts regarding the handling of eggs outside the nest have not been well demonstrated.

Egg retrieval behavior was first observed in greylag geese ( *Anser anser* ), where parent birds used their beak and lower jaw to pull eggs back into the nest from nearby areas (Lorenz and Tinbergen 1970). This behavior has since been widely documented in precocial birds (Lank et al. 1991; Lyon and Shizuka 2017), particularly those nesting on the ground or on water surfaces, often with simple nest structures. For example, this behavior was observed in species such as the American coot ( *Fulica americana* ) (Lyon and Shizuka 2017) and Eurasian stone‐curlew ( *Burhinus oedicnemus* ) (Spena et al. 2019). These species may accidentally displace their eggs from the nest due to high risk of nest predation or intraspecific competition, as their simple nests are prone to disturbance. The presence of eggs outside the nest may be perceived by the parent bird as a potential threat, prompting the evolution of egg retrieval behavior (Petit, Petit, and Petit 1989; Zaun and Weathers 2009; Lank et al. 1991). However, the research on American coots suggests that intraspecific brood parasitism may also play an important role in the evolution of this behavior, where parasitic eggs are retrieved into the nest and subsequently rejected (Lyon and Shizuka 2017).

In the obligate brood parasitism systems, hosts evolved the ability to recognize and reject foreign eggs due to the high costs of parasitism (Rothstein 1990; Davies 2000; Soler 2014). Ground‐nesting or secondary cavity‐nesting hosts are more likely to develop recognition and retrieval behaviors as they could see eggs near the nest cup. Therefore, under parasitic selection pressures such as mislaid cuckoo eggs (Thomson, Tolvanen, and Forsman 2016; Peer and Vozza 2024) or egg‐evicting behavior by parasitic chicks (Kilner and Davies 1999; Kilner 2005), the hosts are more likely to retrieve their own eggs from outside the nest while rejecting most non‐mimetic parasitic eggs (Yang and Zhang 2024). The studies observed egg retrieval behavior in hosts or potential hosts of common cuckoos and cowbirds (Peer and Vozza 2024; Yang et al. 2024; Yang and Zhang 2024). For example, in potential hosts like the green‐backed tit ( *Parus monticolus* ), it was found that parent birds tend to retrieve highly mimetic conspecific eggs while directly rejecting non‐mimetic model eggs (Yang, Liang, and Møller 2019). Similarly, studies on two buntings and prothonotary warblers ( *Protonotaria citrea* ) also revealed that the degree of egg mimicry influences the decision to retrieve or reject eggs outside the nest (Peer and Vozza 2024; Zhang et al. 2024).

Incorrectly retrieving a parasitic egg or mistakenly rejecting own egg can lead to significant reproduction costs (Yang, Liang, and Møller 2019; Peer and Vozza 2024). Therefore, the parasitic pressure (or level of egg mimicry) and the egg recognition ability of the host may influence the handling of eggs outside the nest cup by ground‐nesting or cavity‐nesting hosts. Hosts with low rejection rates tend to retrieve eggs outside the nest cup regardless of mimicry, as seen in studies on prothonotary warblers (Peer and Vozza 2024), varied tit (*Sittiparus varius*) and marsh tit ( *Poecile palustris* ) (Yang et al. 2024), as these species tend to either ignore or retrieve conspecific or non‐mimetic eggs rather than reject them. However, for hosts with high egg rejection rates, two scenarios may occur: if the parasitic egg closely mimics the eggs of the host, the host is likely to retrieve it, mistaking it for its own, or may accept it (or ignore) due to difficulty in distinguishing it. Conversely, if the parasitic egg is poorly mimetic, the host is more likely to reject it. For hosts with strong rejection ability, such as the green‐backed and Japanese tits ( *Parus minor* ), they tend to reject non‐mimetic eggs while retrieving mimetic conspecific eggs (Yang et al. 2024). In theory, when the motivation to retrieve their own eggs conflicts with rejecting parasitic eggs, the hosts may experience decision delays due to the high mimicry of the external eggs, leading to higher rates of ignoring them. To date, high ignoring rates of conspecific eggs were observed only in prothonotary warblers and a population of Japanese tits, despite their opposite rejection rates (Peer and Vozza 2024; Yang et al. 2024). Furthermore, aside from prothonotary warblers and two buntings, other studies have not identified the degree of mimicry of parasitic eggs or even the identity of the parasites (Yang, Liang, and Møller 2019; Yang et al. 2024; Yang and Zhang 2024; Liu et al. 2023). Therefore, further research is needed with appropriate systems to verify the trade‐off of the effect of anti‐parasitic adaptations on the decision‐making process of hosts when handling eggs outside the nest cup.

**FIGURE 1:**
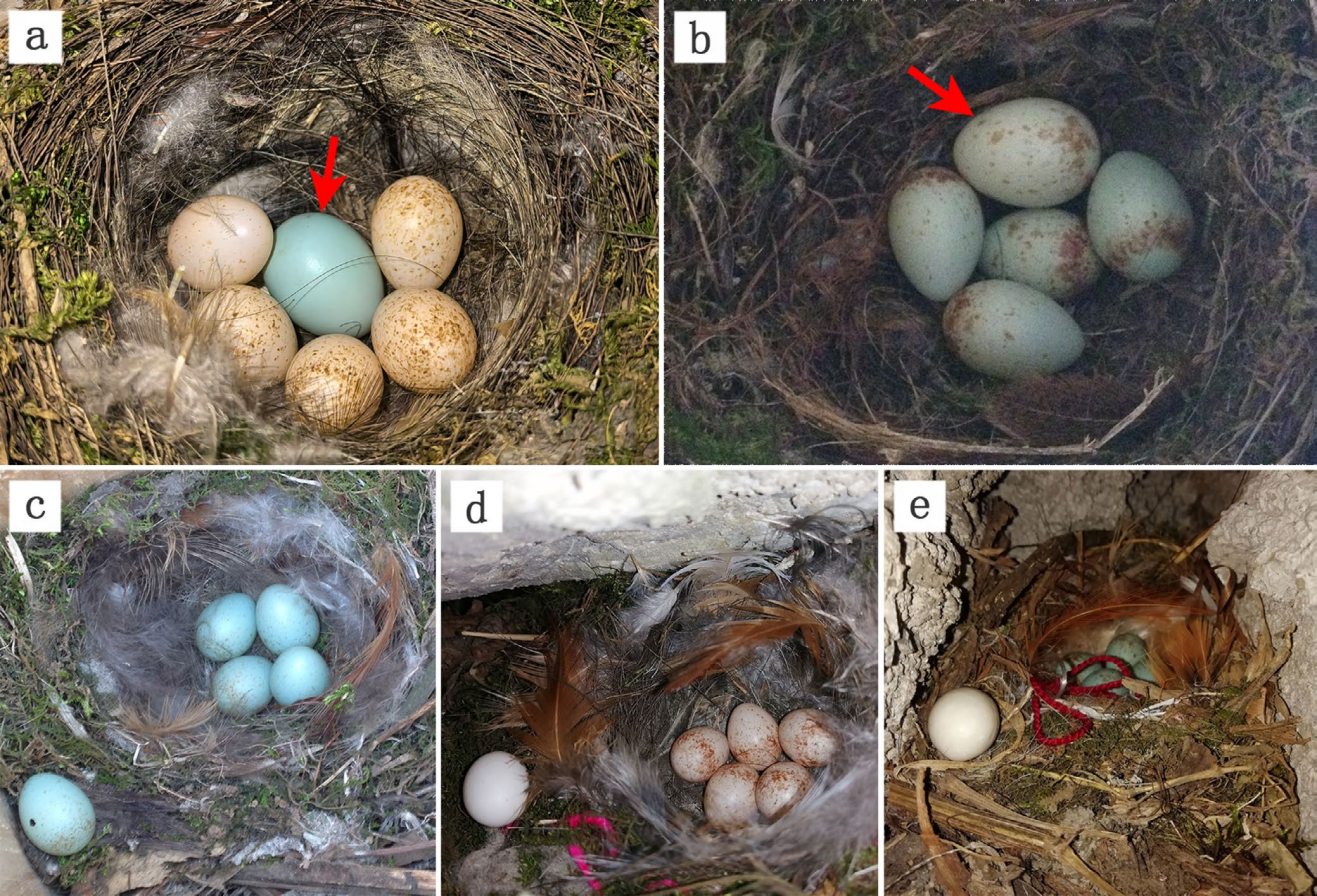
Photographs of the natural and experimental parasitism manipulation. The letters a and b refer to egg‐laying in Daurian redstart nests by common cuckoos, with arrows indicating the cuckoo eggs; letter c refers to a conspecific egg placed on the rim of host nest cup, letter d refers to a budgerigar egg placed on the rim of host nest cup and letter e refers to a white model egg placed on the rim of the host nest cup.

Previous studies showed that common cuckoos parasitizing Daurian redstarts ( *Phoenicurus auroreus* ) lay both mimetic and non‐mimic parasitic eggs (Figure 1a,b). These hosts nest in secondary cavity‐holes or on structures like platforms in buildings and exhibit a high capability for egg recognition, rejecting both non‐mimetic and conspecific eggs with different phenotypes (Yang et al. 2016; Zhang et al. 2021). Due to the restricted entrances of natural nests, cuckoos may occasionally mislay eggs outside the nest cup, and the egg‐evicting behavior of cuckoo chicks may also drive the evolution of egg retrieval behavior in the parent birds.

In this study, we simulated the mislaying of cuckoo eggs outside the nest to explore the cognitive adaptive mechanisms of the Daurian redstart in handling eggs outside the nest cup. If the host exhibits a high rate of ignoring towards mimetic conspecific eggs edge of the nest cup while directly rejecting non‐mimetic eggs, it would indicate that the handing of eggs outside the nest cup is the result of the motivation conflict between rejecting parasitic eggs and retrieving their own eggs. Alternatively, if they tend to ignore or reject the eggs outside the nest cup (whether mimetic or not), it may suggest that the motivation of Daurian redstarts to reject parasitic eggs may outweighs their instinct to retrieve.

## 2 Materials and Methods

### 2.1 Study Site and Species

The current study was conducted from March to August during the years 2019–2022 in the Liuzhi area of Guizhou, Southwest China (26°13′ N, 106°42′ E). The sample area is located within the North Temperate Monsoon climate zone at an elevation ranging from 1070.89 to 1657 m. The region primarily consists of a karst landscape, featuring a mosaic of villages, farmland, scrub forests, and barren slopes (Zhong et al. 2023).

The Daurian redstart is a sexually dimorphic resident bird species, with the breeding season at our study site occurring from late March to early August. They typically nest in cavities or platforms on buildings, road embankments, or slopes, reflecting a strong association with human habitation (secondary cavity nesters). The clutch size ranges from 3 to 6 eggs, commonly 4 or 5, with the female beginning incubation after laying the last egg (Zhong et al. 2019). In this study area, the Daurian redstart is one of the primary hosts of the common cuckoo, which typically lays two types of eggs with varying degrees of mimicry in the dim environments of cavity nests (Figure 1a,b). Previous work showed that Daurian redstarts exhibit a high rejection rate for non‐mimetic model eggs (Yang et al. 2016; Zhang et al. 2021).

### 2.2 Field Experiments

We monitored bird activity for each breeding season in the study area and searched for nesting sites based on their breeding status. Upon locating a host nest, we recorded the egg‐laying progress and conducted experiments during the late egg‐laying or incubation stages (Figure 1c–e). Given the diversity in nesting sites of the Daurian redstart ranging from open to strictly cavity nests, we selected nests with ideal edge space for the following experiments:

1. A conspecific egg was placed 2–3 cm outside the nest cup, with a concealed mark for identification if retrieved (2.11 ± 0.03 g in egg mass, 19.31 ± 0.12 mm × 14.51 ± 0.07 mm in egg size, *N* = 32). These eggs are primarily collected from fresh eggs laid in the early stages of egg‐laying (with only one egg taken from each nest) and from nests that have been abandoned due to interference or destruction by other animals.
2. A white, unspotted model egg was placed at the nest edge (2.51 ± 0.03 g in egg mass, 19.73 ± 0.65 mm × 14.53 ± 0.46 mm in egg size, *N* = 20). The process of making model eggs follows the method of Zhong et al. (2023).
3. To address concerns that artificial egg material may affect the rejection results (Yang et al. 2024), a real white budgerigar ( *Melopsittacus undulatus* ) egg was placed at the outer edge of nest cup (2.28 ± 0.25 g in egg mass, 20.30 ± 0.98 mm × 15.85 ± 0.83 mm in egg size, *N* = 20).

The egg recognition and rejection experiments showed that Daurian redstarts typically reject eggs within 3 days, with most rejections occurring within 24 h (Yang et al. 2016). We examined the status of the experimental eggs on the second and third days after the day of the experiment and monitored some nests with video surveillance (using a Uniscom‐T71 camera, with dimensions 70 × 26 × 12 mm; Mymahdi Technology Co. Ltd., Shenzhen, China). If during the 3‐day experiment period the egg outside the nest cup disappeared or had pecking marks while the eggs inside remained normal, it was recorded as a rejection. If the experimental egg was found inside the nest cup (with highly mimetic conspecific eggs identified by a marking signal), it was recorded as a retrieval. If the experimental eggs remained in its original position outside the nest and had no pecking marks, it was considered ignored (Lyon and Shizuka 2017; Yang, Liang, and Møller 2019).

### 2.3 Statistical Analysis

To compare the handling of different mimetic eggs by the Daurian redstarts outside the nest cup (retrieval, ignoring, and rejection), we conducted statistical analyses using Fisher's exact test or Pearson's chi‐square test in R (R_3.6.3_) (R Core Team 2018). When comparing the differences in certain behaviors of hosts across different experimental groups, we combined the other two behaviors into one for comparison (presence vs. absence). All tests were two‐tailed, with a significance level set at 0.05. Egg parameters are presented as mean ± standard deviation (SD).

## 3 Results

Our experimental results showed that Daurian redstarts retrieved 18.2% of highly mimetic conspecific eggs placed outside the nest cup, while ignoring 60.6% and even rejecting 21.2%. Contrastingly, 75% of unspotted non‐mimetic budgerigar eggs were rejected, with an ignoring rate of 25%. The white model eggs had an even higher rejection rate of 86.4% and an ignoring rate of 13.6% (Table 1). However, no eggs outside the nest cup were retrieved in any of the experimental groups with budgerigar eggs or model eggs. In addition, the video showed that the host is rejecting eggs outside the nest cup through an ejection method (Videos [Link], [Link]).

**TABLE 1:** Behavioral responses of the Daurian redstart ( *Phoenicurus auroreus* ) to three types of experimental eggs placed on the edge of the nest cup.

Experimental egg type	Nest tested (*N*=)	Egg retrieval (%)	Egg ignoring (%)	Egg rejection (%)
Conspecific egg	33	6 (18.2)	20 (60.6)	7 (21.2)
Budgerigar egg	36	0	9 (25)	27 (75)
White model egg	22	0	3 (13.6)	19 (86.4)

The comparison revealed that the ignoring rate for conspecific eggs was significantly higher than that for budgerigar (*ꭓ* ^2^ = 7.557, *df* = 1, *p* = 0.006) and white model (*ꭓ* ^2^ = 10.116, *df* = 1, *p* = 0.001) eggs. However, the rejection rate for conspecific eggs was significantly lower than that for non‐mimetic budgerigar (*ꭓ* ^2^ = 17.835, *df* = 1, *p* < 0.001) and white model (*ꭓ* ^2^ = 19.941, *df* = 1, *p* < 0.0001) eggs. Additionally, the retrieval rate for conspecific eggs was significantly higher than that for non‐mimetic budgerigar eggs (Fisher's exact test, all *p* = 0.009) but not significantly different from model eggs (Fisher's exact test, *p* = 0.071).

## 4 Discussion

The research suggests that the egg retrieval behavior of some ground‐nesting and secondary cavity‐nesting birds near their nests is an important adaptive strategy to prevent offspring loss (Petit, Petit, and Petit 1989; Lank et al. 1991; Yang et al. 2024; Zhang et al. 2024). However, when faced with the cost of mistakenly retrieving parasitic eggs or wrongly rejecting their own eggs, hosts in avian brood parasitism systems may exhibit decision delays or ignoring behaviors due to the difficulty in recognizing highly mimetic eggs. Our study on the Daurian redstart, a host of the common cuckoo, found that it exhibited a higher rejection rate for non‐mimetic eggs (unspotted budgerigar and white model eggs), although it showed a higher ignoring rate for highly mimetic conspecific eggs. This result supports the hypothesis that host decision‐making in handling eggs outside the nest cup in obligate brood parasitism systems is driven by parasitic pressure (Yang, Liang, and Møller 2019; Peer and Vozza 2024). Additionally, the low retrieval rates and rejection selection for conspecific eggs may indicate a strong anti‐parasitic motivation in the Daurian redstart host.

Earlier studies showed that potential hosts, such as the willow tit ( *Poecile montanus* ), coal tit ( *Periparus ater* ), and white‐rumped shama ( *Copsychus malabaricus* ) tend to retrieve conspecific eggs while ignoring non‐mimetic model eggs (Yang et al. 2024; Yang and Zhang 2024). In species like the varied tit, marsh tit, and common pochard ( *Aythya ferina* ), both highly mimetic conspecific and non‐mimetic eggs tend to be retrieved (Hořák and Klvaňa 2009; Yang et al. 2024). Additionally, in the Japanese tit, green‐backed tit, and Oriental magpie‐robin, parent birds show a low ignoring rate for eggs (conspecifics and non‐mimetic model eggs) (Yang, Liang, and Møller 2019; Yang et al. 2024; Liu et al. 2023). However, the Daurian redstarts show a high rate of ignoring rather than retrieving conspecific eggs, although they exhibit a high tendency to directly reject non‐mimetic eggs. Similar results were found in the prothonotary warbler, where the retrieval rate of highly mimetic eggs was low, and the ignoring rate was very high (Peer and Vozza 2024). And they directly rejected non‐mimetic objects both inside and outside the nest (Peer and Vozza 2024). Therefore, our study likely also supports the hypothesis that host retrieval behavior is modified by cognitive adaptations to anti‐parasitism (Yang et al. 2024).

In the Daurian redstart system, common cuckoos lay both mimetic and non‐mimetic eggs, which can complicate egg recognition for the parent birds. This recognition difficulty may lead to decision delays or forced acceptance of highly mimetic conspecific eggs outside the nest. Similarly, in potential hosts with low egg recognition abilities, such as willow tits, coal tits (Yang et al. 2024), and white‐rumped shamas (Yang and Zhang 2024), the high ignoring rate of non‐mimetic eggs should not be classified as egg rejection (because their rejection rate of non‐mimetic eggs outside the nest cup is similar to that inside the nest). Instead, ignoring may more reasonably be considered a form of forced acceptance due to egg recognition limitations.

Additionally, in our experiments with conspecific eggs, we observed a reject rate similar to that of the retrieval rate (18.2% retrieval vs. 21.2% rejection). This phenomenon was not previously reported. We speculate that this may be related to the host's strong anti‐parasitic cognitive adaptations. As a result, even if an external egg closely mimics the own egg of the host, it may be rejected if it is located outside the nest cup. Although conspecific egg rejection occurred in potential hosts like the Japanese tit population (Yang et al. 2024), this behavior may not be directly linked to anti‐parasitic adaptations, as no parasitism cases were reported in these species. Thus, the rejection of external mimetic eggs by Daurian redstarts may indicate that they are currently exhibiting a strong anti‐parasitic cognitive adaptation, specifically over‐recognition.

However, the similar rejection rate of conspecific eggs and their retrieval by Daurian redstarts may also be influenced by other factors such as nest predation and the environment surrounding the nest (Lank et al. 1991; Poláček et al. 2013; Guigueno and Sealy 2017). In this population, Eurasian tree sparrows ( *Passer montanus* ) and Oriental magpie‐robin sometimes prey on or destroy Daurian redstart offspring (Wan et al. 2023), likely due to nest competition among species utilizing secondary cavity‐nests. However, predation typically results in complete destruction of offspring rather than merely removing eggs from the nest edge. Our monitoring showed that the female continued to incubate her eggs normally after the disappearance of the experimental eggs. Therefore, the conspecific eggs should be rejected by the host rather than being due to predation. The reason for this rejection could be influenced by the lighting conditions of the nest and the position of the female during incubation. In dimly lit nests, parent birds may struggle to compare the phenotypes of their own eggs inside the nest with those of the outside, leading to potential misidentification. And since the birds usually only see the eggs outside the nest cup while sitting in the nest, even their own eggs may be mistaken for parasitic ones and rejected. For example, in the ground‐nesting yellow‐throated bunting and south rock bunting with good lighting conditions inside the nest, it was found that they did not reject any simulated conspecific eggs (Zhang et al. 2024). It is theoretically impossible for hosts to distinguish conspecific eggs based solely on signal differences, so rejection of eggs outside the nest cup may stem from their position, leading the parent bird to suspect them as parasitic or due to changes in egg phenotype perception caused by their location. However, due to the complexity of factors affecting the judgment of parasitic eggs, future studies need to explore various aspects to understand the process by which host reject of conspecific eggs outside the nest.

In conclusion, our study on the common cuckoo host Daurian redstart reveals that the decision‐making process for handling retrieval of eggs outside the nest cup is likely influenced by anti‐parasitic rejection motivations driven by parasitism pressure. We propose that the behavior of ignoring eggs outside the nest cup in cuckoo hosts is an adaptive outcome of the conflict between egg retrieval and rejection motivations, possibly related to the hosts' egg recognition difficulties. However, the adaptive trade‐offs in handling eggs outside the nest cup needs to be further validated under different light conditions (open nests and ground nests) and in hosts with varying levels of anti‐parasitism cognition.

## Author Contributions

**Guo Zhong:** formal analysis (lead), investigation (equal), methodology (equal), resources (equal), writing – original draft (equal). **Guixia Wan:** investigation (equal), methodology (equal). **Longwu Wang:** conceptualization (equal), funding acquisition (equal), resources (equal), supervision (equal), writing – review and editing (equal). **Wei Liang:** conceptualization (equal), funding acquisition (equal), supervision (equal), validation (lead), writing – review and editing (equal).

## Ethics Statement

The experiments comply with the current laws of China. Experimental procedures were in accordance with the Animal Research Ethics Committee of Hainan Provincial Education Centre for Ecology and Environment, Hainan Normal University (No. HNECEE‐2012‐004) and Experimental Animal Ethics Committee of Guizhou Normal University (No. 2021001).

## Conflicts of Interest

The authors declare no conflicts of interest.

## Supporting information


**Video S1.** A female Daurian redstart pecking and ejecting a conspecific egg from the rim of its nest cup.

**Video S2.** A female Daurian redstart pecking and ejecting a budgerigar egg from the rim of its nest cup.

**Video S3.** A female Daurian redstart pecking and ejecting a white model egg from the rim of its nest cup.

Table S1.

Table S2.

## Data Availability

Supporting Information (Videos [Link], [Link], [Link]) and data used in this study (Data Tables S1 and S2) are available at https://figshare.com/s/892bed69d8bf3ee94168 (doi: 10.6084/m9.figshare.27109348).
